# Measurement of maternal functioning during pregnancy and postpartum: findings from the cross-sectional WHO pilot study in Jamaica, Kenya, and Malawi

**DOI:** 10.1186/s12884-020-03216-z

**Published:** 2020-09-07

**Authors:** Jenny A. Cresswell, Kelli D. Barbour, Doris Chou, Affette McCaw-Binns, Veronique Filippi, Jose Guilherme Cecatti, Maria Barreix, Max Petzold, Nenad Kostanjsek, Sara Cottler-Casanova, Lale Say

**Affiliations:** 1 Development and Research Training in Human Reproduction (HRP), Department of Sexual and Reproductive Health and Research, UNDP UNFPA UNICEF WHO World Bank Special Programme of Research, World Health Organization, Geneva, Switzerland; 2 Department of Obstetrics and Gynecology, Baylor College of Medicine, Houston, Texas USA; 3 Department of Community Health and Psychiatry, University of the West Indies, Mona, Kingston, Jamaica; 4 Faculty of Epidemiology and Population Health, London School of Hygiene and Tropical Medicine, London, UK; 5 Department of Obstetrics and Gynecology, School of Medical Sciences, University of Campinas, Campinas, Brazil; 6 School of Public Health, Institute of Medicine, University of Gothenburg, Gothenburg, Sweden; 7 Department of Information, Evidence and Research, World Health Organization, Geneva, Switzerland; 8 Swiss Tropical and Public Health Institute (Swiss TPH), Basel, Switzerland; 9 University of Basel, Basel, Switzerland

**Keywords:** WHODAS, maternal health, health-related functioning, health-related quality of life, activities of daily living

## Abstract

**Background:**

The World Health Organization’s definition of maternal morbidity refers to “a negative impact on the woman’s wellbeing and/or functioning*”*. Many studies have documented the, mostly negative, effects of maternal ill-health on functioning. Although conceptually important, measurement of functioning remains underdeveloped, and the best way to measure functioning in pregnant and postpartum populations is unknown.

**Methods:**

A cross-sectional study among women presenting for antenatal (*N* = 750) and postpartum (*N* = 740) care in Jamaica, Kenya and Malawi took place in 2015–2016. Functioning was measured through the World Health Organization Disability Assessment Schedule (WHODAS-12). Data on health conditions and socio-demographic characteristics were collected through structured interview, medical record review, and clinical examination. This paper presents descriptive data on the distribution of functioning status among pregnant and postpartum women and examines the relationship between functioning and health conditions.

**Results:**

Women attending antenatal care had a lower level of functioning than those attending postpartum care. Women with a health condition or associated demographic risk factor were more likely to have a lower level of functioning than those with no health condition. However, the absolute difference in functioning scores typically remained modest.

**Conclusions:**

Functioning is an important concept which integrates a woman-centered approach to examining how a health condition affects her life, and ultimately her return to functioning after delivery. However, the WHODAS-12 may not be the optimal tool for use in this population and additional components to capture pregnancy-specific issues may be needed. Challenges remain in how to integrate functioning outcomes into routine maternal healthcare at-scale and across diverse settings.

## Background

In 2015, an estimated 303 000 women died during or after pregnancy [1]. Despite the global focus on maternal mortality, deaths represent only a small fraction of the total burden of ill-health due to inadequate maternal healthcare [2]. The World Health Organization (WHO) estimates that, in 2016 alone, maternal conditions contributed to around 19 million disability-adjusted life years lost [3]. Indirect maternal conditions, such as preexisting hypertension and mental health conditions, are becoming increasingly important and have important implications for long-term health across the life course [4, 5]. This is especially the case for settings experiencing an obstetric transition with a marked decrease of direct causes of maternal morbidity and mortality in parallel with improvement in economic development, a situation often found in middle-income countries [6]. However, maternal morbidity has been inconsistently and poorly defined and measured, thus the total burden remains unclear.

In 2018, the WHO Maternal Morbidity Working Group (MMWG) published a new conceptual framework, the Maternal Morbidity Measurement (MMM) Framework, to promote better measurement of maternal morbidity and highlight the importance of using a woman-centred approach [5]. This MMM Framework is aligned with the standardised definition of maternal morbidity and associated disability developed by the same group: *any health condition attributed to and/or complicating pregnancy and childbirth that has a negative impact on the woman’s wellbeing and/or functioning”* [7]. Maternal health is a social and economic phenomenon, not just a clinical issue [8], and the context and environment in which a woman and her family live has an important effect on outcomes, as recognised by the current strategy towards ending preventable maternal mortality (EPMM) [9]. The MMM Framework recognises that optimal maternal health is not limited to a single short-span event; and considers a life-cycle approach to better reflect how previous pregnancies and experiences impact current and future pregnancy outcomes.

The MMWG has previously published a matrix of health conditions that identify cases of maternal morbidity [7], however the latter part of the definition, namely “… *that has a negative impact on the woman’s wellbeing and/or functioning*”, requires further development and testing. Conceptually, the inclusion of wellbeing and/or functioning is important as it recognises the significance of woman’s experience of ill-health, and how any health condition affects her life and, by implication, that of her family and community for an unlimited period of time, alongside her productive and non-productive capabilities [10]. Qualitative work has shown that many women prioritise practical concerns around how their health condition affects their daily functioning, which may not align closely with the emphasis often placed by healthcare providers [11]. This is often an under-explored area: a systematic review of reviews found that the majority of outcomes in trials of intrapartum interventions focused on adverse events rather than reflecting positive health and well-being [12]. Furthermore, Sustainable Development Goal (SDG) 3, which aims to “ensur…[e] healthy lives and promot…[e] the well-being for all at all ages”, along with many other SDGs including SDG-5 on gender equality, SDG-8 on decent work and economic growth, and SDG-10 on reduced inequalities, will not be met without commitment to addressing the holistic needs of pregnant and childbearing women.

A substantial number of studies have documented the, mostly negative, effects of maternal ill-health on functioning, using a variety of assessment tools [8, 13–15]. Nonetheless, extensive challenges remain as to how to operationalise the measurement of wellbeing and/or functioning in pregnant and postpartum populations in practice. First, this population is relatively young and on average relatively healthy, whereas many tools for measuring functioning and disability are targeted at older adults and/or groups with specific health conditions: with implications for the questions and domains covered to ensure they capture both what is important to this population, as well as ability to capture change over a short time period. Second, the state of pregnancy and childbirth is a transient period associated with many biological and social changes, disentangling what is a “normal”, “typical” or “expected” change due to the temporal state of pregnancy versus a change associated with a health condition is not straightforward. Third, there are difficulties in eliciting cross-cultural equivalence of concepts and measurement of wellbeing and functioning, resulting in difficulties in using one uniform tool [16]. In order to measure progress towards reducing maternal morbidity holistically, tools that can measure wellbeing and functioning in a way that can be integrated into routine care and in diverse settings will be needed.

Wellbeing and functioning are related, but distinct concepts. Wellbeing is a subjective measure of how satisfied people are with their lives, health and day to day experiences. Functioning is the positive inverse correlate of disability and is conceptualised by the International Classification of Functioning, Disability and Health (ICF) [17]. The ICF classifies functioning and disability into three levels: the body or body part, the whole person, and the whole person in a social context. It encompasses a person’s ability to perform a task or activity in the environment in which they live. In this study the focus is on the measurement of functioning; wellbeing was not measured.

A pilot study using the WHO-MMWG standardised tool to measure maternal morbidity (the WOICE tool) took place in 2015 and 2016 [18, 19]. The 12-item version of the WHO Disability Assessment Schedule (WHODAS 2.0) [20] was included to document functioning. WHODAS 2.0 has been administered in almost 100 countries and almost 50 languages [21]. It covers six domains: cognition, mobility, self-care, getting along, life activities, and participation. The objectives of this paper are to (1) assess differences in the distribution of functioning levels, as measured by the WHODAS-12, between antenatal and postpartum care; (2) to describe the relationship between poorer functioning and health condition among pregnant and postpartum women.

## Methods

### Data collection

The protocol for the pilot study has been described in detail elsewhere [18], as have the characteristics of the sample [19]. In brief, two cross-sectional samples of women (one antenatal, one postpartum) were selected from public health facilities in Jamaica (6 health centres, 3 referral hospitals), Kenya (2 district hospitals, 1 referral hospital), and Malawi (1 referral hospital). Data collection took place between July 2015 and February 2016. Each country was expected to recruit 250 antenatal and 250 postpartum women. This sample size allowed for a 6% margin of error without pooling data across sites [18].

Participants were a convenience sample of women who gave informed consent presenting for routine antenatal and postpartum care who were at least 28 weeks gestation in the case of the antenatal sample, or were 6–12 weeks postpartum in the postpartum care sample. Written informed consent was obtained, except in cases where the woman was illiterate in which case a witness confirmed the accurate reading of the consent form and a thumbprint of the participant was obtained. This procedure was approved by the ethics committees. Women were eligible to participate regardless of whether their pregnancy ended in a live or still birth.

Data were collected via a structured questionnaire (available to view online: http://maternal-voices.srhr.org/) administered via a tablet. The process consisted of a patient interview, a medical record review for laboratory test results and a physical examination. In Jamaica and Kenya the interviewers were health facility staff (nurses, midwives or doctors), while Malawi recruited nurse-midwives for the study who conducted the physical exam and recent medical graduates conducted the interview.

Ethical approval was obtained from the WHO Ethical Review Committee, the North East Regional Health Authority Jamaica (NERHA), the University of the West Indies Ethics Committee, the South East Regional Health Authority Jamaica (SERHA), the Kenya Medical Research Institute Scientific and Ethics Review Unit (KEMRI), the Malawi College of Medicine Research and Ethics Committee (COMREC).

### Variable definitions

Our primary outcome was the woman’s WHODAS-12 total score. The WHODAS is a set of 12 questions where women are asked about the degree of difficult they have had due to health conditions averaging over the past 30 days [22]. Respondents are asked to take into account how they usually do a given activity when rating the difficulty, for example if they usually use an assistive device [22]. The simple scoring method was used in this study: that is, the sum of the total of the woman’s responses: “none” (0) “mild” (1) “moderate” (2) “severe” (3) and “extreme” (4). The WHODAS questions were asked in the middle of the questionnaire, after the socio-demographic information and initial patient history, but before the clinical exam. There were no missing values. We also graphically presented our distribution alongside the normative data from Australia published by Andrews et al. [23]. In order to describe characteristics associated with reduced functioning, we created quintiles of the WHODAS total score values, separately for the antenatal and postpartum groups, and collapsed the first four quintiles to allow us to compare the least-well functioning 20% of women to the rest of the sample. We chose this approach as we had little existing information as to the relationship between functioning and outcomes and wished to avoid imposing assumptions so far as possible.

Health conditions were recorded as open-ended questions and then coded according to the ICD-10 categories by a clinician (KDB). Direct maternal conditions were hypertensive disorders of pregnancy, obstetric haemorrhage, pregnancy-related infection and other obstetric complications [7]. Indirect maternal conditions were those not classified as direct but aggravated by the physiological effects of pregnancy [7].

Other variables used in this analysis were anxiety score (General Anxiety Disorder, 7-item (GAD-7); depression score (Personal Health Questionnaire, 9-item (PHQ-9); exposure to violence (women who responded no or never to the following questions: (1) Are you afraid of your current/most recent husband or partner or anyone else? Would you say never, sometimes, many times, most/all of the time?; (2) Since pregnancy/delivery, was there ever a time when you were pushed, slapped, hit, kicked, or beaten by (any of) your husband/partner(s) or anyone else?); substance use (reports use of one or more of the following since pregnancy/delivery: tobacco products, alcoholic beverages, marijuana, inhalants); maternal age (in years); previous births (defined as prior to the index delivery); maternal education (highest level attended); marital status (has current partner or not); employed (worked in the last 12 months); and for the postpartum sample: mode of index delivery (vaginal or caesarean); and currently breastfeeding (on day of interview).

### Statistical analysis

Descriptive data (percentiles and percentages) are presented to show the distribution of WHODAS total scores separately among the antenatal and postpartum samples. The crude association between least-well functioning quintile and health condition was assessed using Pearson chi-squared statistic corrected for clustering at the facility-level and converted into an F-statistic. The characteristics associated with least-well functioning were assessed using a logistic regression model with robust standard errors to allow for clustering at the facility-level and adjusted for country of data collection. Country of data collection, direct maternal condition, indirect maternal condition and maternal age were included in the multivariable models *a priori* due to their recognised importance on functioning, other variables were included in the multivariable model if the crude association was P < 0.1. Cronbach’s alpha was calculated to describe internal consistency separately for antenatal and postpartum populations.

## Results

In total, 750 women were interviewed in the antenatal sample (253 from Jamaica, 258 from Kenya, and 239 from Malawi) and 740 women were interviewed in the postpartum sample (256 from Jamaica, 242 from Kenya, 242 from Malawi). Overall, 500 (33.6%) were nulliparous prior to the index pregnancy; 401 (26.9%) had 1 previous birth and 589 (39.5%) had 2 or more previous births. In nearly all of the postpartum sample (*N* = 734; 99.2%), the index pregnancy resulted in a live birth; only 6 women (0.8%) had a stillbirth and 4 (0.5%) infants had died since the delivery. Socio-demographic characteristics of the sample are described in Tables 1 and 2.

**Table 1:** Characteristics associated with the least-well functioning quintile in the antenatal sample (*N* = 750)

		Distribution in Sample N (%)	Model A*			Model B **		
			**OR**	**95% CI**	**p**	**OR**	**95% CI**	**P**
**Country setting**								
Country	Jamaica	253 (33.7%)	1.00		0.0025	1.00		0.0456
	Kenya	258 (34.4%)	1.10	0.56, 2.15		2.00	0.88, 4.57	
	Malawi	239 (31.9%)	0.67	0.36, 1.25		1.27	0.57, 2.83	
**Health condition and associated risk factors**								
Direct maternal condition^a^	None	613 (81.7%)	1.00		0.0933	1.00		0.0398
	1 + direct condition	137 (18.3%)	1.46	0.93, 2.30		1.69	1.03, 2.76	
Indirect maternal condition^a^	None	615 (82.0%)	1.00		0.2618	1.00		0.5607
	1 + indirect condition	135 (18.0%)	1.29	0.81, 2.08		1.18	0.65, 2.13	
Any condition	None	500 (66.7%)	1.00		0.0077			
	1 + condition	250 (33.3%)	1.42	1.12, 1.80				
Anxiety score^b^ [mean (SD)]		2.6 (3.0)	1.22	1.11, 1.35	0.0005	1.16	1.03, 1.32	0.0214
Depression score^c^ [mean (SD)]		2.4 (3.3)	1.19	1.12, 1.28	0.0001	1.10	1.00, 1.22	0.0582
Exposure to violence	No	654 (87.2%)	1.00		0.0807	1.00		0.8435
	Yes	96 (12.8%)	1.63	0.93, 2.86		1.05	0.61, 1.80	
Substance use	No	720 (96.0%)	1.00		0.8182			
	Yes	30 (4.0%)	0.90	0.33, 2.44				
**Socio-demographic characteristics**								
Maternal age	< 20 years	91 (12.1%)	1.00		0.1950	1.00		0.0589
	20–34 years	591 (78.8%)	1.71	0.93, 3.12		2.04	1.17, 3.54	
	≥ 35 years	68 (9.1%)	2.96	0.87, 10.06		3.80	1.16, 12.45	
Previous births	No previous births	260 (34.7%)	1.00		0.2622			
	1 previous births	202 (26.9%)	1.01	0.70, 1.47				
	2 + previous	288 (38.4%)	1.50	0.90, 2.50				
Maternal education	Primary or less	202 (26.9%)	1.00		0.2880			
	Secondary	370 (49.3%)	1.05	0.77, 1.43				
	Tertiary	178 (23.7%)	1.66	0.87, 3.17				
Marital status	No partner	218 (29.1%)	1.00		0.8335			
	Has partner	532 (70.9%)	1.06	0.60, 1.87				
Employed	No	357 (47.6%)	1.00		0.4821			
	Yes	393 (52.4%)	1.20	0.70, 2.06				

* Adjusted for country

** Adjusted for country, direct maternal condition, indirect maternal condition & maternal age a priori, in addition to characteristics associated with p < 0.1 in Model A

^a^As per the ICD-10 classification

^b^Attainable range: 0 to 21

^c^Attainable range: 0 to 27

**Table 2:** Characteristics associated with the least-well functioning quintile in the postpartum sample (*N* = 740)

		Distribution in Sample N (%)	Model A*			Model B **		
			**OR**	**95% CI**	**p**	**OR**	**95% CI**	**P**
**Country setting**								
Country	Jamaica	256 (34.6%)	1.00		< 0.0001	1.00		< 0.0001
	Kenya	242 (32.7%)	3.11	1.83, 5.27		7.01	3.85, 12.75	
	Malawi	242 (32.7%)	0.41	0.25, 0.067		0.86	0.50, 1.49	
**Health condition and associated risk factors**								
Direct maternal condition^a^	None	676 (91.4%)	1.00		0.0148	1.00		0.1188
	1 + direct condition	64 (8.7%)	2.31	1.21, 4.39		1.78	0.84, 3.77	
Indirect maternal condition^a^	None	646 (87.3%)	1.00		0.0043	1.00		0.0304
	1 + indirect condition	94 (12.7%)	2.58	1.43, 4.67		1.96	1.08, 3.55	
Any condition	None	951 (79.9%)	1.00		0.0001			
	1 + condition	149 (20.1%)	2.82	1.88, 4.22				
Anxiety score^b^ [mean (SD)]		1.5 (2.4)	1.22	1.09, 1.36	0.0018	1.11	0.98, 1.27	0.0875
Depression score^c^ [mean (SD)]		1.2 (2.0)	1.30	1.16, 1.46	0.0003	1.19	1.01, 1.40	0.0353
Exposure to violence	No	659 (89.1%)	1.00					
	Yes	81 (11.0%)	1.32	0.67, 2.59	0.3939			
Substance use	No	717 (96.9%)	1.00		0.9225			
	Yes	23 (3.1%)	0.93	0.21, 4.10				
**Socio-demographic characteristics**								
Maternal age	< 20 years	115 (15.5%)	1.00		0.0929	1.00		0.0960
	20–34 years	550 (74.3%)	1.17	0.70, 1.94		1.21	0.68, 2.14	
	≥ 35 years	75 (10.1%)	2.03	1.01, 4.10		2.11	0.97, 4.58	
Previous births	No previous births	240 (32.4%)	1.00		0.3285			
	1 previous	199 (26.9%)	0.81	0.61, 1.07				
	2 + previous	301 (40.27)	0.89	0.55, 1.44				
Maternal education	Primary or less	214 (28.9%)	1.00		0.1719			
	Secondary	356 (48.1%)	1.66	0.87, 3.18				
	Tertiary	170 (23.0%)	1.68	0.85, 3.00				
Marital status	No partner	227 (30.7%)	1.00		0.9286			
	Has partner	513 (69.3%)	1.02	0.69, 1.49				
Employed	No	384 (51.9%)	1.00		0.1707			
	Yes	356 (48.1%)	1.27	0.89, 1.82				
**Characteristics associated with index delivery**								
Mode of delivery	Vaginal	577 (78.0%)	1.00		0.0447	1.00		0.0653
	Caesarean	163 (22.0%)	2.19	1.2, 4.68		2.08	0.95, 4.55	
Currently breastfeeding	No	19 (2.8%)	3.34	0.93, 12.01	0.0478	3.19	0.60, 16.97	0.0401
	Yes	711 (96.1%)	1.00			1.00		
	Infant not alive	10 (1.4%)	4.61	1.23, 17.19		3.10	1.30, 7.40	

* Adjusted for country

** Adjusted for country, direct maternal health condition, indirect maternal health condition & maternal age a priori, in addition to characteristics associated with p < 0.1 in Model A. Any health condition not included in model due to over-adjustment

^a^As per the ICD-10 classification

^b^Attainable range: 0 to 21

^c^Attainable range: 0 to 27

### I) The relationship between functioning and time during pregnancy/postpartum

**Fig. 1:**
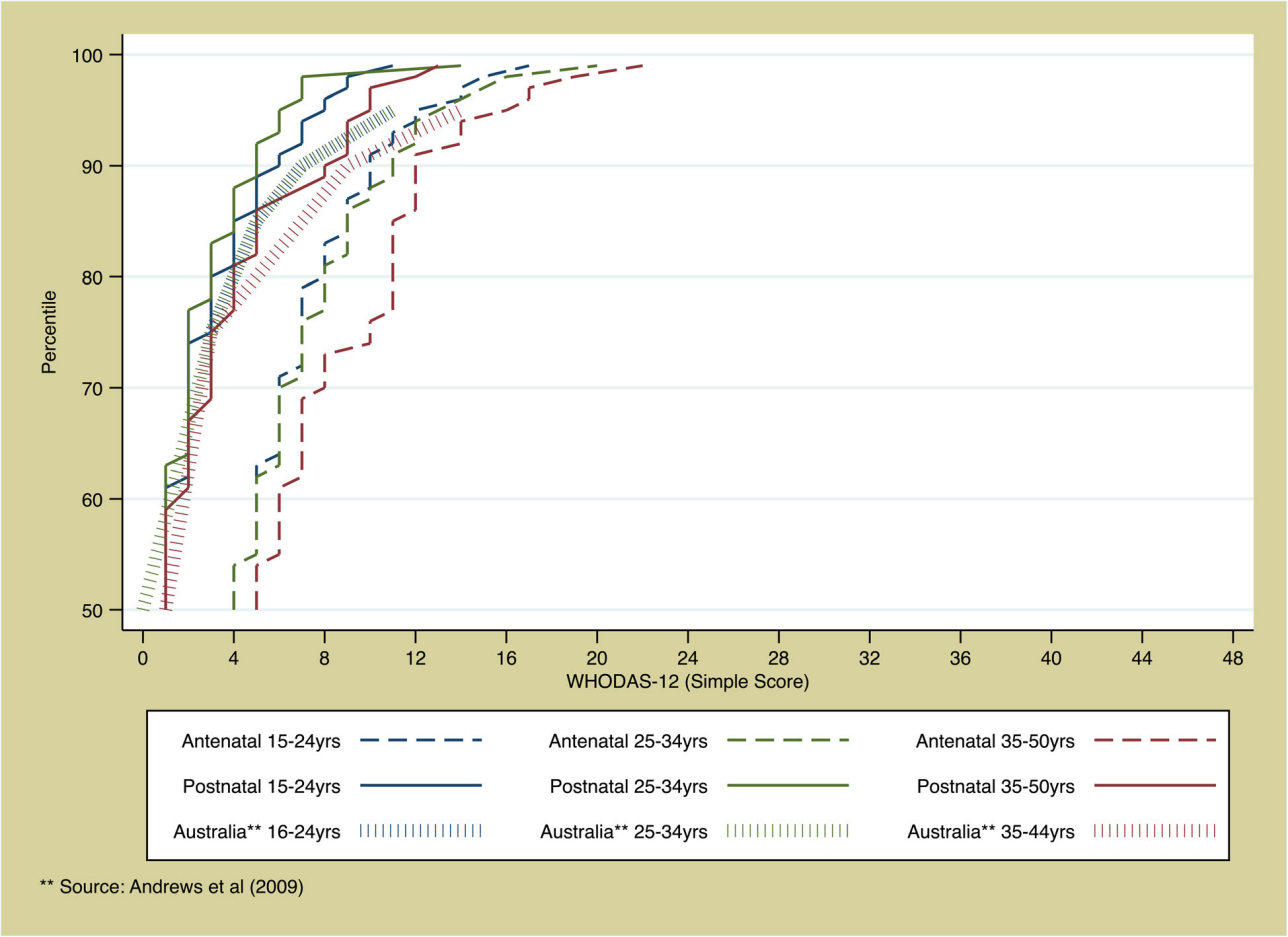
Distribution of WHODAS scores

The distribution of functioning scores is presented in Fig. 1. During the antenatal period, women had a mean WHODAS total score of 5.0 (SD: 4.2), a median score of 4 (IQR: 2, 7) with a range of 0 to 23. Cronbach’s alpha was 0.79 in the antenatal sample. Approximately one in ten (N = 89; 11.9%) women scored zero indicating they reported no functional limitations at all. The cut-off for the 90th percentile was 11, which would correspond to a woman reporting “mild” or “moderate” difficulties for most questions. Functioning was lower in the antenatal period than the postpartum period. During the postpartum period, women had a mean WHODAS total score of 1.8 (SD: 2.8), a median score of 1 (IQR: 0, 3) with a range of 0 to 25. Cronbach’s alpha was 0.77 in the postpartum sample. Nearly half (*N* = 342; 46.2%) of women scored zero; whilst the cut-off for the 90th percentile was 5.

**Fig. 2:**
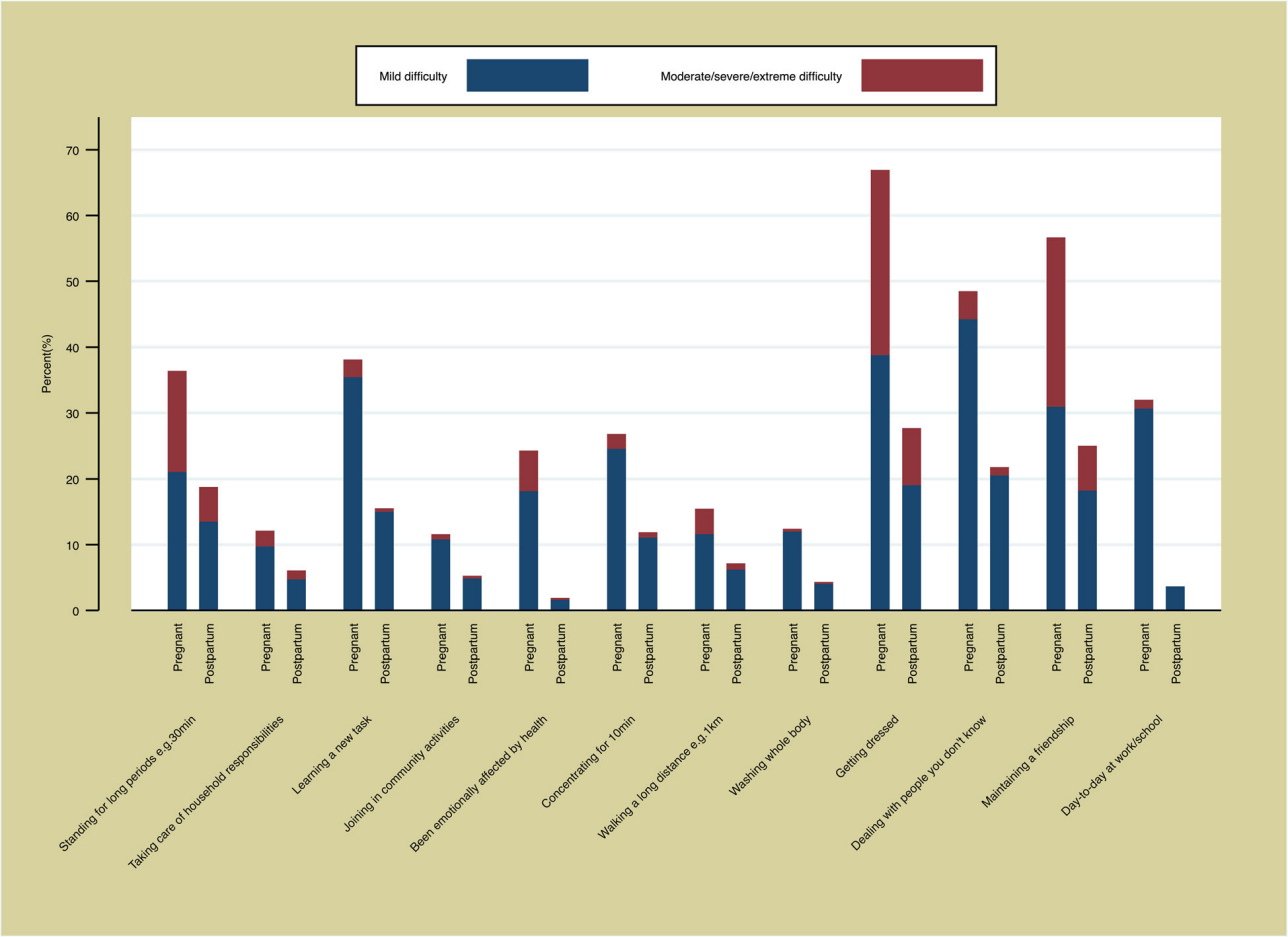
Breakdown of responses for the WHODAS-12

Figure 2 presents the specific areas where women reported the most difficulties in carrying out activities. During pregnancy, women primarily reported moderate or greater difficulties with the mobility domain (walking a long distance, standing for long periods) and life activities (taking care of household responsibilities, day-to-day work/school), whereas relatively fewer women reported difficulties with the more cognitive items (learning a new task, concentrating) or getting along (dealing with people you don’t know, maintaining a friendship). However, it should be noted that even on the highest scoring items a clear majority were reporting none or mild difficulties. During postpartum, very few women reported difficulties in any area, those that did primarily mobility related.

### II) The relationship between functioning and health condition

In the antenatal sample, 250 women (33.3%) had at least one clinical diagnosis of a health condition (115 direct-only; 113 indirect-only; 22 both direct and indirect); in the postpartum sample 149 women (20.1%) had a clinical diagnosis (55 direct-only; 85 indirect-only; 9 both direct and indirect).

Table 3 presents the distribution of women between those with a health condition and those with impaired functioning. There was strong evidence of association between poor functioning and having one or more health condition during both antenatal (*p* = 0.0059) and postpartum (*p* = 0.0003) care. This association remained after adjusting for maternal age (antenatal: *p* = 0.0059; postpartum: *p* = 0.0004). Nonetheless, there was a sizeable group of women in both populations who either had no diagnosed health condition and poor functioning (e.g. 16% and 15% respectively) or did have a health condition and were not in the least-well functioning group (78% and 68% each). No particular health condition was dominant.

**Table 3:** Cross-tabulation between health condition and poor functioning

	Antenatal			Postpartum		
	**Quintiles 1–4**	**Least-well functioning quintile (Q5)**	**Total**	**Quintiles 1–4**	**Least-well functioning quintile (Q5)**	**Total**
**No health condition**	418 (83.6%)	82 (16.4%)	500 (100%)	500 (84.6%)	91 (15.4%)	591 (100%)
**1 + health condition**	196 (78.4%)	54 (21.6%)	250 (100%)	101 (67.8%)	48 (32.2%)	149 (100%)
**Total**	614 (81.9%)	136 (18.1%)	750 (100%)	601 (81.2%)	139 (18.8%)	740 (100%)
***P*-value**	0.0059			0.0003		

The characteristics associated with the least-well functioning quintile are presented in Tables 1 and 2 for antenatal and postpartum care respectively. In general, for both the antenatal and postpartum samples, factors related to health condition and associated risk factors showed stronger evidence of an association compared to socio-demographic characteristics. In both the antenatal and postpartum samples, women in Kenya were more likely to be in the least-well functioning quintile, compared to women from Jamaica or Malawi; this was especially true during the postpartum period. There was weak evidence of a large effect between increased maternal age and being in the least-well functioning quintile.

## Discussion

In this pilot study, we observed a lower level of functioning during pregnancy relative to the postpartum period. Women with a clinically diagnosed health condition were generally more likely to have a lower level of functioning compared to those with no condition. The distribution of scores between the postpartum sample and a general female sample from Australia were similar (Fig. 1), with the exception of the extreme values at the least-well functioning end of the spectrum being less extreme, which may be in part due to a selection effect analogous to a “healthy pregnant woman effect” (women suffering from ill-health may be less likely to become pregnant than their healthier counterparts). Among the antenatal population, there was a shift to the right in terms of the distribution. In both the antenatal and postpartum samples internal consistency, as measured by Cronbach’s alpha, was acceptable.

Our findings are consistent with previous studies showing reduced functioning around the time of childbirth, particularly associated with ill-health. Cross-sectional data from an urban sample of women in the third trimester found a mean WHODAS score of 13.0 in Ghana and 11.8 in Cote d’Ivoire, and that WHODAS score was not associated with physical health factors such as haemoglobin level, but mental health (anxiety and depression) was associated with a significant loss of functioning [24]. A prospective cohort study in Ethiopia found a median WHODAS score of 2 (IQRL 0 ,7) during the third trimester of pregnancy compared to a median WHODAS score of 0 (IQR: 0, 3) in the first two months postpartum [25]. There is evidence that women experience significant changes to their physical and/or mental health and wellbeing between pregnancy and the postpartum periods [26–29]. Timing relative to the birth is important, and the effect may vary according to setting. A prospective cohort study from Malaysia showed that women who experienced severe morbidity had lower overall functional ability (measured using the Inventory of Functional Status after Childbirth) at one month postpartum compared to women without severe morbidity and that the difference between the two groups had disappeared by six months postpartum although most women had not achieved full functional status by this time [14]. Longitudinal qualitative data has found that even among women who deliver healthy full-term infants in a high-income setting, recovery from childbirth; furthermore and resumption of usual activities may take more than six months postpartum [30–32]. A further important issue to be considered is whether the pregnancy ends in a live birth or a still birth.

In our study, the differentials in functioning were relatively small in magnitude. The median WHODAS score of 4 in the antenatal population is approximately equivalent to a small number of responses indicating “mild” difficulties, whilst the 90th centile score of 11 is approximately equivalent to “mild” or “moderate” for most of the twelve items. In part, this is because the sample was predominantly women with an uncomplicated delivery or non-severe morbidity. This is in contrast with a retrospective cohort study in Brazil that recruited women who had experienced a severe complication and concluded that maternal morbidity negatively impacted postpartum functioning up to five years later [15, 33]. However, it should be noted that in the Brazilian study many events, including other pregnancies, are likely to have occurred in the interim.

Many of the women who reported poor levels of functioning also screened positive for anxiety and/or depression. Maternal mental health is an important issue that warrants further attention [34]. In many settings, primary care services rarely screen for mental health problems during pregnancy or the puerperium and the data from this study indicate the need for further research to explore the effectiveness of efforts to sensitize community health teams to the mental health challenges women face, and ways in which mental health interventions could be integrated into routine care.

It is possible that the WHODAS-12 is not the optimal tool to measure changes in functioning in this population group. There are clear advantages to using a tool known to produce reasonably cross-culturally appropriate measures to allow comparisons across time and place and different groups. Nonetheless, an additional module that allowed for functioning specifically relevant to pregnant and postpartum populations may be needed [13]. Most of the reduced functioning reported by women in this study was related to the questions on mobility (standing and walking) followed by life activities (such as household work and work outside the home). Whereas – because the WHODAS is designed to be non-diseases specific – there is no opportunity to ask questions that may be highly relevant such as infant care responsibilities [14]. In our postpartum group, we observed a strong association between reduced functioning and failure to breastfeed, in some instances due to perinatal loss, another domain that data suggest is particularly relevant [35, 36]. There are tools, such as the Mother-Generated Index (MGI) for assessing postpartum quality of life (wellbeing); it allows for the index items to be chosen by the women themselves based on what she feels is important [37]. However, the integration of a tool such as the MGI into routine maternal and newborn care would be very challenging due to the time and resources required, in addition to challenges in interpretation across settings – which is particularly important for WHO due to its role to lead global norms and standards. Administration of the MMWG tool took approximately 45 to 65 min for the administration of the tool, including 15 to 25 min for the physical exam [18].

Our study was exploratory in nature and had a number of limitations. Importantly, the data are cross-sectional. We were unable to truly investigate changes in women’s functioning in line with the MMWG definition, given the constraints imposed by the type of study. Most important are the lack of longitudinal follow-up to allow us to assess change and lack of an adequate control population representing women of reproductive age before conception. Second, the data were selected though health facilities in a non-probability-based sample and thus the results cannot be generalised to the general population in these settings. Third, errors during data collection mean that we do not have adequate data on gestational age and were unable to control for this in our analyses, although all women in the antenatal sample were in the third trimester. Fourth, we do not have data available on wellbeing: the other parallel concept in the WHO maternal morbidity definition and also relevant for the post-2015 agenda [38]. Fifth, we did not conduct any parallel qualitative investigations, so are unable to comment on whether women report their functioning differently according to culture and environment. Sixth, there is the potential for assessment bias, as typically the same person recorded the data on the participants reported health and functioning, and the physical exam. This mirrors what would usually happen during a routine consultation, and our aim is to generate a tool that can be integrated into routine care. In practice, we believe the risk of assessment bias influencing the findings reported in this study is relatively low, as the interviewers were not familiar with the WHODAS-12 and would be unlikely to mentally calculate the scale during their physical examination.

One outstanding issue that it is important to explore further in order to develop a tool optimised to pregnant and postpartum populations is the question of what is “normal”? The WHODAS, for example, asks women to use the last 30 days as their frame of reference, but for this group changes in health and functioning may be taking place very rapidly (particularly in the 3rd trimester), as may expectations and coping mechanisms [11]. It is also a time when substantial changes in typical or usual activities are expected, particularly if the birth is the woman’s first child. Further qualitative work to investigate the thought process women are making when answering these questions would be helpful. Such work would also involve refinement of the essential domains relevant to pregnant and postpartum populations, as discussed above.

## Conclusions

We observed a lower level of functioning during pregnancy relative to the postpartum period. Women with a clinically diagnosed health condition were generally more likely to have a lower level of functioning compared to those with no condition. Functioning is an important concept which integrates a woman-centered approach to examining how a health condition affects her life, and ultimately her return to functioning after delivery. However, the WHODAS-12 may not be the optimal tool for use in this population and additional components to capture pregnancy-specific issues may be needed. Going forward, it will also be important to identify interventions that can be used to reduce functioning impairments in pregnant and postpartum women [11, 39–41]. Potential strategies include providing targeted information, referrals to work, improvement and better adaptation of working conditions, health and social services as needed, and widening the scope of maternal health services. However, in order to design and evaluate interventions in an evidence-based way it is first necessary to clearly define and operationalise women’s functioning during pregnancy and the postpartum period.

## Data Availability

The datasets analysed during the current study are not publicly available due to the inclusion of individual-level patient data; the corresponding author can be contacted in order to facilitate approval for data sharing with the country teams on a case by case basis upon reasonable request.

## Abbreviations

WHO: World Health Organization
MMWG: Maternal Morbidity Working Group
MMM: Maternal Morbidity Measurement Framework
ICF: International Classification of Functioning, Disability and Health
WHODAS: World Health Organization Disability Assessment Schedule
NERHA: North East Regional Health Authority Jamaica
SERHA: South East Regional Health Authority Jamaica
KEMRI: Kenya Medical Research Institute Scientific and Ethics Review Unit
COMREC: Malawi College of Medicine Research and Ethics Committee
ICD: International Classification of Diseases
GAD: Generalized Anxiety Disorder
PHQ: Patient Health Questionnaire
